# A Rare Case of Recurrent Renal Infarcts With Unique Etiologies in Different Kidneys Occurring Six Years Apart

**DOI:** 10.1155/crin/8233593

**Published:** 2024-12-04

**Authors:** Kaitlyn Perkins, Emilyn Anderi, Mariam Costandi, Karla D. Passalacqua, Katarzyna Budzynska

**Affiliations:** ^1^Department of Family Medicine, Henry Ford Hospital, Detroit 48202, Michigan, USA; ^2^Department of Graduate Medical Education, Henry Ford Hospital, Detroit 48202, Michigan, USA

## Abstract

Renal infarcts are uncommon, difficult to diagnose, and can lead to long-term kidney disease. Because they have numerous etiologies and patients may present with nonspecific symptoms, renal infarcts may be mistaken for other common conditions. A 50-year-old woman presented to the emergency department (ED) with flank pain, nausea, and vomiting. Computed tomography (CT) revealed multiple right kidney infarcts, transthoracic echocardiography revealed mitral valve stenosis with no evidence of atrial fibrillation, and hypercoagulability tests were negative. High-intensity anticoagulation therapy resolved the infarcts and she was discharged on warfarin. Six years later, at the age of 56, the woman again presented to the ED with back pain, nausea, vomiting, and fever. She had undergone valvuloplasty to repair the mitral valve stenosis 1 month before this ED visit, and warfarin had been discontinued shortly after the procedure. CT imaging and ultrasonography showed no evidence of infarcts and electrocardiogram was normal. Although urinalysis was negative for infection, pyelonephritis was suspected per CT results. However, renal function and leukocytosis did not improve after 2 days of antibiotic therapy. Radioisotope renal scan then revealed infarcts in the left kidney. Anticoagulation therapy again led to recovery, and the patient was discharged back on warfarin. After the recurrent infarct, monitoring and cardiac care have led to adequate long-term management, and no evidence of atrial fibrillation has ever been observed. This case illustrates the challenging diagnosis of an unusual presentation of recurrent renal infarct, where each infarct was suspected to have a unique and independent etiology: mitral valve stenosis in the first and hypercoagulability from withdrawal of warfarin in the second. Because no clear risk or symptom profiles exist for renal infarcts, this unusual condition should be considered when patients do not respond to treatment for other renal problems, especially those with cardiovascular disease.

## 1. Introduction

Renal infarction is rare and usually results from an abrupt reduction of the arterial blood supply to the kidney. The true prevalence of renal infarcts is not completely known; however, one autopsy study estimated the incidence to be 1.4% [1], while a more recent study of 250,000 patients seen in the emergency department (ED) over 4 years reported a rate of only 0.007% [2]. Diagnosing renal infarcts is difficult because it is a rare condition and patients may present with nonspecific symptoms such as abdominal or flank pain, nausea, vomiting, and fever; thus, patients can easily be misdiagnosed with other more common conditions such as nephrolithiasis or pyelonephritis, which can lead to delayed diagnosis and lack of timely care [3]. Critically, because renal infarcts are challenging to identify, missed diagnoses can lead to serious lifelong consequences, such as chronic kidney disease and end-stage renal disease [4].

The most common underlying causes of renal infarcts are cardioembolic disease, hypercoagulable states, in situ renal artery thrombosis, and direct renal artery injury such as dissection [5], although idiopathic cases have been reported [6]. Atrial fibrillation is a common cardiac risk factor for renal infarcts, and myocardial infarction and rheumatic mitral stenosis are other notable cardiac causes [7–9]. Additionally, underlying injury from renal trauma, Marfan syndrome, and polyarteritis nodosa may lead to renal infarcts [5]. And for patients with no identifiable cardiac concerns who are suspected of having renal infarct, hypercoagulability should be assessed, with the workup including Factor V Leiden, antithrombin III activity, and proteins C and S [3, 5].

Here we present a patient who had 2 rare occurrences of renal infarction that occurred in different kidneys in episodes and were 6 years apart. While the first renal infarcts in the right kidney, which caused long-term kidney damage, were attributed to mitral stenosis, the true cause of the second episode cannot definitively be determined, but most likely occurred from withdrawal of anticoagulation therapy.

## 2. Case Presentation

**Figure 1:**
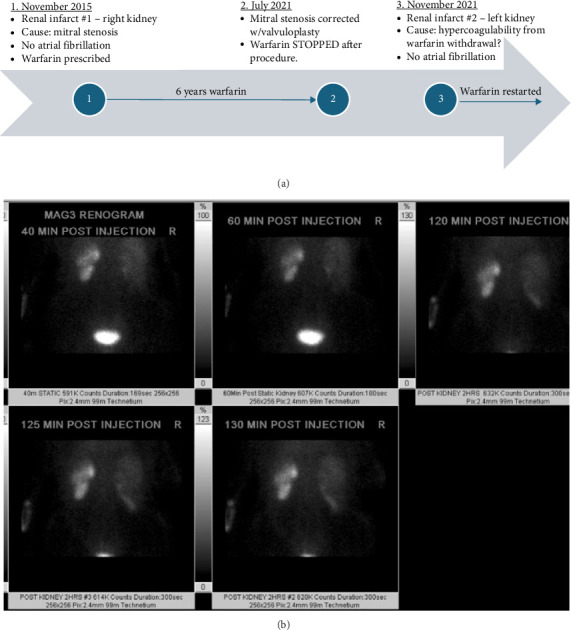
(a) Timeline of key events between two presentations with renal infarcts in different kidneys. (b) Nuclear medicine mercaptoacetyltriglycine (MAG3) renal scan. This scan shows a borderline enlarged left kidney with multiple wedge-shaped peripheral photopenic defects highly suggestive of infarcts, less likely for severe pyelonephritis. Scarring is thought unlikely without parenchymal loss. Abbreviations: HRS, hours; MIN, minute; sec, seconds.

A woman in her 6th decade of life had two ED presentations within 6 years of each other in which renal infarcts in different kidneys were identified (Figure 1(a)). During her first ED visit in 2015, the patient was 50 years old and had been experiencing right-sided flank pain, nausea, and vomiting for 2 days. Shortly before arrival at the ED, she developed severe (10/10) right flank pressure radiating to her midline back. The patient's significant medical history at this time included epilepsy and depression. Her vital signs were blood pressure 149/89 mmHg; pulse 76 beats/min; temperature 36.5°C; body mass index 27.4 kg/m^2^; and SpO_2_ 99%. A physical examination was essentially normal, with a negative abdominal exam, a soft, nondistended abdomen, and tenderness to palpation in the right flank without rebound or guarding. Liver function tests were mildly elevated, lipase was negative, magnesium was slightly low at 1.6 mg/dL and repleted, urinalysis was negative for urinary tract infection, coagulation tests were normal, and lactate was initially 2.7 mmol/L, which decreased to 1.8 mmol/L after a 1 L normal saline bolus. Lactate dehydrogenase (LDH) was elevated at 508 IU/L on initial lab work and increased to 1658 IU/L one day later (reference range (RR) < 250 IU/L).

During this first ED presentation, CT of the abdomen and pelvis showed no evidence of aortic intramural hematoma or dissection but did reveal a pattern of alternating bands of nonenhancement in the right kidney with no perirenal fat stranding or hydronephrosis. The primary differential diagnosis was multiple right renal infarcts, with other less likely considerations including acute pyelonephritis or ureteric obstruction. A broad workup was performed to investigate the source of the infarcts, and transthoracic echocardiography revealed a stenotic, rheumatic mitral valve but no evidence of atrial fibrillation. Cardiology specialists were consulted, and rheumatology specialists performed an autoimmune workup. Coagulability test results including Factor V Leiden and methylenetetrahydrofolate reductase (MTHFR) 677C > T variant were both negative. High-intensity anticoagulation therapy was started for the renal infarcts, and because the patient had nightly fevers and a rheumatic valve, empiric antibiotics for possible endocarditis were also started. The patient's symptoms resolved within 1 week of anticoagulation therapy, and she was continued on antibiotics for 5 more days per infectious disease recommendations. She was started on daily warfarin (10–15 mg), which she took for the next 6 years, over which time she received care through nephrology, hematology, and cardiology.

In 2021, 6 years after the initial ED presentation, at the age of 56, the patient once again presented to the ED, this time with 2 days of progressively worsening abdominal and back pain, nausea, vomiting, and fever. Importantly, 1 month before this ED visit, the patient had received balloon valvuloplasty via the right femoral vein to correct her rheumatic mitral stenosis, and she had stopped taking warfarin shortly after the valvuloplasty procedure. Warfarin was stopped because physicians felt she no longer had any indication for anticoagulation due to the corrected mitral valve stenosis and because the patient had no evidence of atrial fibrillation.

Physical examination in the ED revealed a high temperature of 37.9°C but no signs of injury. Palpations of the abdomen revealed soft tissue with shifting dullness, hepatomegaly, splenomegaly, or mass, as well as abdominal tenderness in the left lower quadrant with left costovertebral tenderness. Initial laboratory tests done in the ED revealed poor kidney function, with high creatinine at 6.06 mg/dL (RR < 1.16), low glomerular filtration rate at 8 mL/min/1.73 m^2^ (RR > 60 mL/min/1.72 m^2^), high potassium of 6.1 mmol/L (RR 3.5–5.0 mmol/L), high white blood cell count of 17.2 K/*μ*l (RR 3.8–10.6 K/*μ*l), and elevated hemoglobin at 15.2 g/dL (RR 12.0–15.0 g/dL). LDH was high at 1007 IU/L (RR < 250 IU/L). Urinalysis was negative for infection and showed proteinuria (100 mg/dL) with moderate blood. COVID-19 test was negative: She had no known history of COVID-19 and had received 2 doses of the COVID-19 vaccine approximately 6 months before this ED presentation (about 2-3 months before the valvuloplasty). Electrocardiogram showed normal sinus rhythm with rightward axis, confirming a lack of atrial fibrillation. Within about 1 h of intravenous sodium chloride bolus, potassium lowered to within RR at 4.6 mmol/L, and creatinine was still high (5.97 mg/dL), although decreasing.

The combination of symptoms including fever, leukocytosis, acute renal failure, and location of pain was suggestive of infectious processes such as pyelonephritis or diverticulitis. CT of the abdomen and pelvis with contrast showed severe right renal atrophy and perinephric fat stranding around the left kidney, further supporting the possibility of pyelonephritis, but no calculi, hydronephrosis, or signs of infarction or diverticulitis were seen. Thus, she was admitted to the medical floor for suspected pyelonephritis and acute renal failure and was given empiric intravenous ceftriaxone (2 g). However, urinalysis returned negative for infection and positive for hematuria, supporting a diagnosis of nephrolithiasis.

After 2 days, the patient had no improvement in renal function or leukocytosis. Renal ultrasonography with arterial duplex showed mild heterogeneity of the left kidney with no hydronephrosis and no significant stenosis of the renal arteries. Based on the patient's recent discontinuation of warfarin, lack of improvement on antibiotics, and history of renal infarction, recurrent renal infarct was considered. The nephrology team put the patient on heparin, and hematology specialists were consulted to assess a possible thrombophilic state. Notably, hematology tests comprising antithrombin III activity and presence of prothrombin genetic risk variants were negative, suggesting that genetic hypercoagulability and thrombophilia were not present.

To further investigate the possibility of renal infarction, a radioisotope renal scan was performed, which showed multiple wedge-shaped peripheral photopenic defects of the left kidney, highly suggestive of infarcts (Figure 1(b)). Also, the patient's atrophic right kidney was displaying minimal function. At this point, the cause of the left kidney infarcts was suspected to be thromboembolic or atheroembolic due to the recent catheterization procedure and discontinuation of warfarin; however, given the patient's previous mitral stenosis, underlying cardioembolic causes were also possible. Nephrologists discussed the possibility of future kidney transplantation, but the patient's kidney function continued to improve over 8 days. After a total of 8 days in the hospital, heparin therapy was discontinued and she was discharged back on daily warfarin (10 mg) with plans to follow up with electrophysiologists for cardiac function testing.

At follow-up 6 weeks later, cardiologists gave the patient a 28-day Holter monitor, which revealed at least 2 episodes of possible intermittent and sudden loss of conduction for 7 and 3 s, with the latter pause lacking P waves; however, motion artifacts limited interpretation of these results. Therefore, considering these possible cardiac events and the unpredictability of conduction loss, cardiologists suggested implanting a pacemaker or loop recorder to monitor for possible atrial fibrillation, which could have been an underlying cause for the recurrent infarcts. The patient then sought a second opinion with another cardiologist, who also recommended pacemaker placement; however, the patient did not want pacemaker placement, and she was directed to monitor herself for specific symptoms, such as syncope and presyncope. The patient ultimately had a loop recorder implanted approximately 7 months after her hospital discharge. Over the course of 1 year, the loop recorder did not reveal any arrhythmias, and the patient has since had the loop recorder removed. The patient has been on warfarin and continued regular follow-up care with her primary care physician, cardiologist, and nephrologist with no further infarctions or complications.

## 3. Discussion

Here we described an unusual case of a patient who had 2 recurring episodes of renal infarcts that were identified 6 years apart and occurred in different kidneys; notably, the causes of these infarcts were unique, complex, and most likely multifactorial. The first infarction in the right kidney, which led to long-term kidney damage, had been confidently attributed to the presence of mitral stenosis, since the patient had tested negative for hypercoagulability at the time. Interestingly, atrial fibrillation was not a suspected cause of the first infarcts because the patient had normal sinus rhythm per multiple electrocardiograms. However, her second renal infarct occurred shortly after her mitral stenosis had been corrected and after she had stopped anticoagulation therapy. Therefore, the second infarct event may have been caused by a thromboembolic event from the recent cessation of warfarin; however, a definitive etiology for the second occurrence cannot be definitively known, and considering the patient's cardiac condition, atrial fibrillation was also thought to be a causative factor, although no evidence of atrial fibrillation had ever been detected.

Renal infarction may be overlooked because no clear risk profile for this rare occurrence has been determined. At the time of diagnosis, most patients with renal infarct are older and have discernible cardiac disease, most often atrial fibrillation [6, 10], whereas patients with idiopathic renal infarct are usually younger and have fewer cardiovascular risk factors [6]. One retrospective study observed that 10% of patients with idiopathic renal infarct were eventually diagnosed with atrial fibrillation during follow-up, and older patients with provoked renal infarct were more likely to have recurrence [10]. Reports of recurrent renal infarct are particularly sparse and include one patient who had protein S deficiency and one patient who had been using anabolic steroids [11, 12]. Additionally, COVID-19 may be yet another element in the risk for renal infarction, since one patient who lacked all other thrombotic risk factors developed renal infarct during SARS-CoV-2 infection [13]. Our patient had no history of COVID-19, but she had been vaccinated about 6 months before her ED presentation. While coagulation complications associated with vaccines have been observed, to our knowledge, only 1 vaccine-related occurrence of renal infarction after vaccination (AstraZeneca ChAdOx1) has been reported, and this occurred only 13 days after vaccination [14]. An important element of our patient's trajectory was that her second renal infarction was initially thought to be pyelonephritis, which aligned with her general symptoms and initial CT imaging results that curiously did not reveal the left kidney infarcts; nonetheless, the patient's history of kidney disease, cardiac disease, and her age made renal infarction a possibility when infection became a less likely scenario, and this situation was confirmed by renal radioisotope imaging. Thus, because no obvious risk profile exists for renal infarction, physicians should keep this possibility on the differential diagnosis for both younger and older patients who have recalcitrant kidney dysfunction, particularly for those with atrial fibrillation, previous instances of renal infarct, a history of hypercoagulability, and perhaps recent COVID-19 or COVID-19 vaccination.

No specific recommended therapeutic strategies have been established for the immediate management of renal infarction, but depending upon the severity and suspected underlying cause, physicians may initiate conservative care with anticoagulation therapy up through surgical revascularization [15, 16], and a full discussion of the many schools of thought regarding immediate treatment are beyond the scope of this report. However, a critical long-term factor in caring for patients who have had renal infarct is managing underlying cardiovascular conditions when present and monitoring for progressive kidney disease. One single-center retrospective study showed that 33.8% of patients with a history of renal infarct went on to develop chronic kidney disease [17], and another study observed that about half of patients with renal infarct developed renal impairment while 6.5% developed severe impairment [10]. Our patient developed chronic kidney disease 3b/A2 after her first renal infarct, which worsened to G4/A2 after her second one, and hypertension and acute kidney injury may also have contributed to her worsening kidney health. Therefore, patients who experience renal infarct should have kidney function tested regularly and receive appropriate counseling for best kidney health practices.

Our patient currently receives follow-up care with multiple specialists, takes several medications, works full time, and is a mother. Extensive counseling and consistent follow-up have been key to the successful and ongoing management of her chronic disease, and we recommend that physicians take into account each patient's unique and multifaceted life situation when caring for patients who could develop worsening renal disease. Overall, this case highlights the complexity of renal infarct development, the difficulty of discerning infarcts from other more common infectious causes of kidney dysfunction, and the importance of vigilantly monitoring renal and cardiac function in patients with a history of concomitant heart and kidney disease so that timely treatment can be given to avoid long-term renal impairment from infarction.

Renal infarct, especially recurring infarction, is extremely uncommon and difficult to diagnose; however, early recognition and prompt treatment are critical for minimizing the risk of organ damage, and close follow-up is essential to ensure that patients do not develop serious negative long-term consequences from this unusual and unpredictable renal event. In particular, patients with a history of any combination of cardiac disease, kidney disease, anticoagulation therapy, and potentially COVID-19 or COVID-19 vaccination who are presenting with symptoms of pyelonephritis should be assessed for renal infarcts.

## Data Availability

Data sharing is not applicable to this article as no new data were created or analyzed in this study.
